# A fatal case of repeated ventricular fibrillation due to torsade de pointes following repeated administration of metoclopramide

**DOI:** 10.1002/ccr3.6213

**Published:** 2022-08-09

**Authors:** Yusuke Watanabe, Itaru Nakamura, Yasuyuki Takebayashi, Hidehiro Watanabe

**Affiliations:** ^1^ Department of Infection Prevention and Control Tokyo Medical University Hospital Tokyo Japan

**Keywords:** arrhythmia, metoclopramide, QT prolongation, renal failure, torsade de pointes

## Abstract

Although metoclopramide has many adverse effects, torsade de pointes (TdP) is rare. We describe a fatal case of repeated ventricular fibrillation due to TdP following repeated administration of metoclopramide. Administration of multiple doses of metoclopramide over a short time to a patient with risk factors for TdP should be avoided.

## Graphical abstract

Although metoclopramide has many adverse effects, torsade de pointes (TdP) is rare. Repeated administration of metoclopramide in combination with risk factors for TdP may cause ventricular fibrillation following TdP.

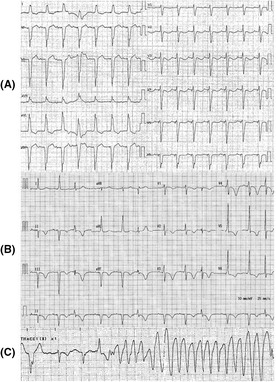

## 1 INTRODUCTION

Metoclopramide is a dopamine 2‐receptor antagonist with antiemetic activity that causes release of acetylcholine from postganglionic nerve endings and prevents apomorphine‐induced vomiting.^1^ Metoclopramide is an antiemetic agent used in chemotherapy and to treat post‐operative nausea and vomiting.^2^, ^3^ Although metoclopramide has many adverse effects,^1^ torsade de pointes (TdP) is not stated.^4^ Risk factors of TdP include advanced age, female sex, congestive heart failure, and myocardial infarction, corrected QT (QTc) >500 ms, QT‐prolonging drugs, impaired hepatic drug metabolism, hypokalemia, hypomagnesemia, hypocalcemia, diuretics causing hypokalemia and hypomagnesemia, and bradycardia.^5^ Recently, the findings of a meta‐analysis have suggested that metoclopramide increases cardiac event risk, including sudden cardiac death.^6^ However, few case reports have linked TdP with metoclopramide, and none have reported fatalities. We describe a fatal case of repeated ventricular fibrillation (VF) due to TdP following administration of multiple doses of metoclopramide in combination with risk factors for TdP.

## 2 CASE REPORT

**FIGURE 1:**
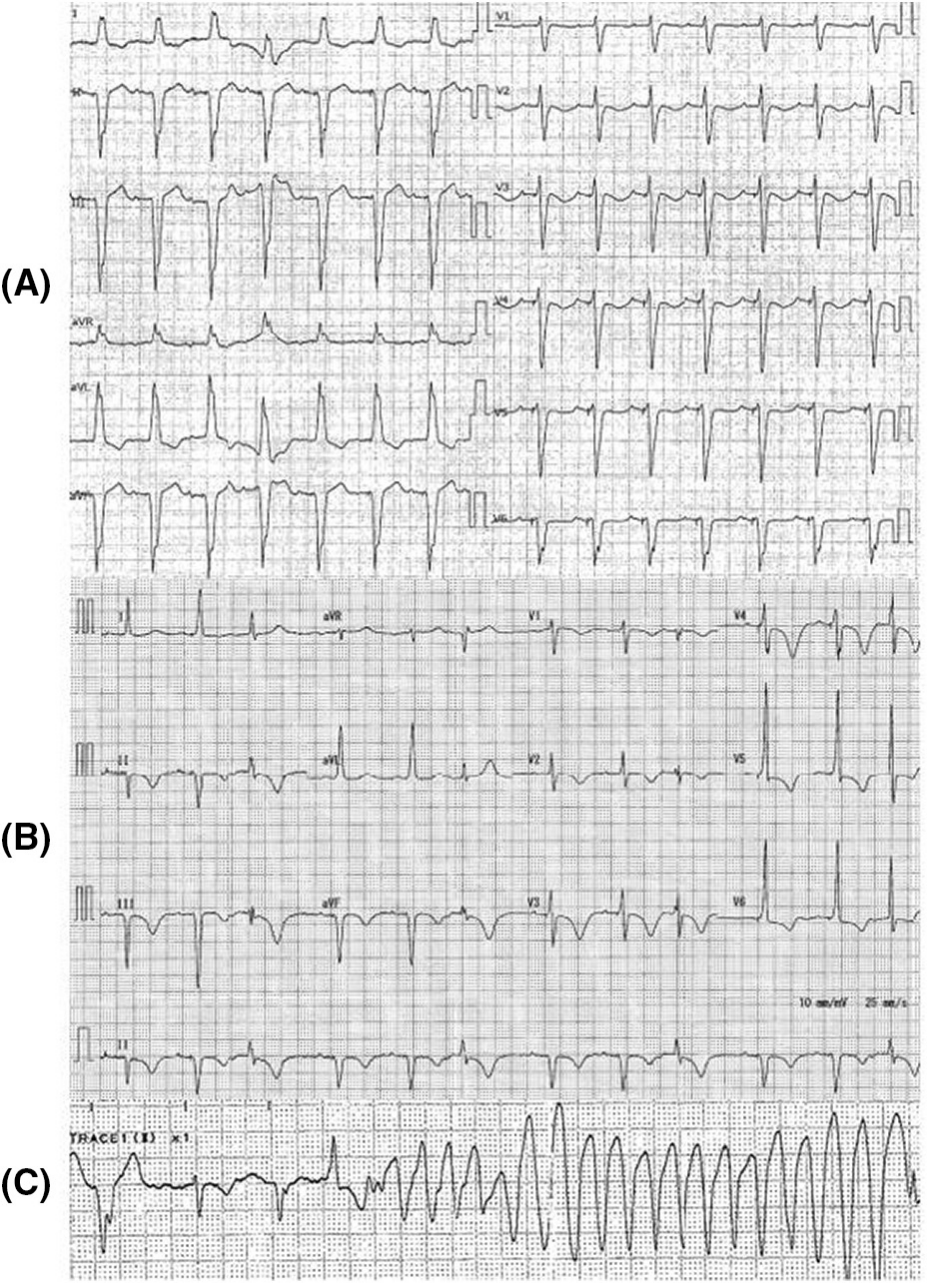
(A) The electrocardiogram on admission shows a corrected QT of 453 ms. (B) The electrocardiogram 9 hours after initial administration of metoclopramide shows a corrected QT of 551 ms. (C) The onset of torsade de pointes 20 h after the initial administration of metoclopramide.

A 92‐year‐old woman was admitted for epigastric pain, nausea, and diarrhea that persisted for 1 week. Her medical history consisted of a complete atrioventricular block on a DDD pacemaker with basic rate 60 bpm and upper rate response 130 bpm, atrial fibrillation, ischemic cardiac disease, and chronic kidney disease. She had been taking enalapril, spironolactone, azosemide, carvedilol, apixaban, rabeprazole, rosuvastatin, nicorandil, and zolpidem. On admission, her body temperature was 36.7°C, blood pressure 109/75 mmHg, heart rate 58 bpm, and oxygen saturation 98% on room air. She reported slight tenderness on percussion in the right hypochondriac region. The laboratory data showed leukocytes 20,600/μl, aspartate aminotransferase 27 U/L, alanine aminotransferase 41 U/L, creatinine 2.07 mg/dl, potassium concentration 4.0 mmol/L, magnesium concentration 1.8 mg/dl, and C‐reactive protein 25.9 mg/dl. Her electrocardiogram (ECG) revealed QTc interval of 453 ms (Figure 1A). Her echocardiogram showed mild diffuse hypokinesis with an ejection fraction of 45%, mild‐to‐moderate mitral regurgitation, mild‐to‐moderate aortic valve stenosis, left ventricular end‐diastolic diameter of 46 mm, left ventricular end‐systolic diameter of 34 mm, left atrial diameter of 39 mm, interventricular septum thickness of 12 mm, and posterior left ventricular thickness of 11 mm. There was no change compared with 1 week earlier. She received three drip infusions of metoclopramide 10 mg for nausea after admission, and 9 h after initial administration, ECG showed QTc prolongation of 551 m/s (Figure 1B). She suddenly lost consciousness with TdP 20 h after initial administration (Figure 1C). At that time, laboratory analysis showed the following: CK‐MB 7 U/L, troponin T 0.088 ng/dl, potassium concentration 5.1 mmol/L, corrected calcium concentration 9.9 mg/dl, and magnesium concentration 1.8 mg/dl. Venous blood gas analysis revealed pH 7.344, oxygen partial pressure 37.9 mmHg, carbon dioxide partial pressure 40.3 mmHg, bicarbonate 21.5 mmol/L, and lactate 2.08 mmol/L. Contrast CT after initial Tdp stopped revealed a liver abscess and portal vein thrombosis. Despite cardiopulmonary resuscitation with three defibrillation attempts, one for every instance of VF following TdP, and administration of adrenalin and amiodarone, VF returned. Magnesium infusion, isoproterenol infusion, and overdrive pacing were not performed because the VF rapidly followed TdP. We discontinued resuscitation after a total of three defibrillations attempts because her family did not wish for more resuscitation, and the patient died. No antibiotics were administrated. Her blood culture was positive for *Escherichia coli* on the fifth day after collection.

## 3 DISCUSSION

QT prolongation caused by metoclopramide has been reported in some studies.^7^, ^8^, ^9^ A recent study stated that supratherapeutic doses of metoclopramide might lead to QT and action potential prolongation by inhibiting the human ether‐a‐go‐go‐related gene and sodium channels, resulting in the development of ventricular arrhythmias.^8^ Moreover, 80% of metoclopramide is excreted in the urine, and impaired renal function prolongs its half‐life.^1^ Therefore, we hypothesized that QT prolongation due to metoclopramide occurred due to the administration of multiple doses in a patient with impaired renal dysfunction.

To our knowledge, there have only been three surviving cases of TdP associated with metoclopramide administration (Table 1). All cases experienced TdP caused by metoclopramide and had other risk factors. The first case was a 92‐year‐old woman taking two different QT‐prolonging drugs,^10^ the second case was an 86‐year‐old man who was administered metoclopramide four times daily with heart and renal failure,^11^ and the third case was a 50‐year‐old woman taking three different QT‐prolonging drugs.^12^ Although our patient had been taking diuretics, her electrolytes were almost normal. None of the patient's other drugs interacted with metoclopramide, and she was not taking any QT‐prolonging drugs known to cause TdP.^4^ Therefore, three administrations of 10 mg metoclopramide in addition to her advanced age, female sex, ischemic cardiac disease, and chronic kidney disease might have contributed to repeated VF following TdP.

**TABLE 1:** Characteristics of three cases of torsade de pointes associated with metoclopramide

Age (years)	Sex	Underlying disease	Dose of metoclopramide	Medication	QTc (ms)	Treatment	Outcome
92	F	HIV	70 mg for 3 days	erythromycin cisapride	653	magnesium and lidocaine were administered	lived
86	M	heart failure renal failure	10 mg 4 times daily	none	597	defibrillation	lived
50	F	bradycardia	single dose of 10 mg	methadone metronidazole	460	defibrillation potassium and magnesium were administered	lived

Abbreviations: M, male; F, Female; HIV, human immunodeficiency virus.

Information on sudden cardiac death from metoclopramide is limited. A case–control study reported that the adjusted odds ratio of metoclopramide for sudden cardiac death compared to no exposure was 4.31.^13^ A retrospective cohort study reported that the risks of all‐cause and cardiovascular mortality were higher among new users of metoclopramide compared with domperidone, which also increased the risk of sudden cardiac death.^14^, ^15^ A study from the Clinical Practice Research Datalink in the United Kingdom reported that the odds ratio of sudden cardiac death with exposure to metoclopramide compared with non‐exposure ranged from 4.31 to 4.93.^16^ Therefore, although there are a few case reports on cardiac events caused by metoclopramide, the risk of sudden cardiac death following TdP due to QT prolongation may be increased.

Intravenous magnesium is a first‐line therapy, and maintaining potassium levels has been recommended in patients with TdP.^17^ Magnesium is used empirically to prevent ventricular arrhythmia in the patients with normal or low magnesium levels.^18^ Because the patient in our case had normal magnesium levels and almost normal potassium levels, magnesium infusion was a treatment option.

Metoclopramide may promote QT prolongation and lead to VF following TdP. Administration of multiple doses of metoclopramide over a short time to a patient with risk factors for TdP should be avoided.

## AUTHOR CONTRIBUTIONS

YW, YT, and IN were involved in the clinical management. YW wrote the manuscript. IN and HW was involved in the revision of the manuscript. All authors approved the final version for submission.

## CONFLICT OF INTEREST

The authors declare that they have no conflicts of interest to disclose.

## ETHICAL APPROVAL

Ethical approval was not required. Informed consent was obtained from the patient.

## CONSENT

Written informed consent was obtained from the patient to publish this report in accordance with the journal's patient consent policy.

## Data Availability

The data that support the findings of this study are available from the corresponding author upon reasonable request.
